# Assessment of a Voluntary Non-Profit Health Insurance Scheme for Migrants along the Thai–Myanmar Border: A Case Study of the Migrant Fund in Thailand

**DOI:** 10.3390/ijerph16142581

**Published:** 2019-07-19

**Authors:** Nareerut Pudpong, Nicolas Durier, Sataporn Julchoo, Pigunkaew Sainam, Beena Kuttiparambil, Rapeepong Suphanchaimat

**Affiliations:** 1 International Health Policy Program (IHPP), the Ministry of Public Health, Nonthaburi 11000, Thailand; 2 Dreamlopments Ltd., Bangkok 10310, Thailand; 3 UNICEF Thailand Country Office, Bangkok 10200, Thailand; 4 Department of Disease Control, Bureau of Epidemiology, the Ministry of Public Health, Nonthaburi 11000, Thailand

**Keywords:** health protection, health insurance, migrant health, undocumented migrant, illegal migrant, migrant, Migrant Fund, M-Fund

## Abstract

Access to health care and financial protection for migrants can be promoted through diverse health insurance schemes, designed to suit migrants’ needs within a specific context. The Migrant Fund (M-Fund) is a voluntary, non-profit health insurance scheme operating along the Thai–Myanmar border in Thailand since 2017 and aims to protect the health of migrants uncovered by existing government insurance schemes. A qualitative evaluation was conducted between December 2018 and March 2019 to determine M-Fund’s operational impacts, provide recommendations for improvement, and draw suggestions about its role in protecting migrant health. In-depth interviews with 20 individuals and 5 groups were conducted in three categories: (1) International, national, and local partners; (2) M-Fund clients; and (3) M-Fund staff. Interview information was triangulated with findings from other informants, a document review, and researchers’ observations. Despite covering a small number of 9131 migrants, the M-Fund has contributed to improving access to care for migrants, raised awareness about migrant health protection, and reduced the financial burden for public hospitals. The M-Fund acts as a safety-net initiative for those left behind due to unclear government policy to protect the health of undocumented/illegal migrants. Despite clear merits, the issue of adverse selection to the scheme is a critical challenge. Evidence from this evaluation is useful to inform the future design of government insurance schemes for migrants.

## 1. Introduction

Micro-insurance is accepted as a useful type of insurance for protecting vulnerable groups of people through payments that are tailored to health needs, incomes, and levels of risk [1]. Several countries have introduced micro-insurance in the form of community-based health insurance (CBHI) schemes, to promote access to healthcare and ensure financial protection for the target population, particularly those in poor rural communities [2,3,4]. A wide range of health insurance schemes can be designed to protect the health of migrants within a given country-specific context. For example, the Government of Mexico introduced a cross-border health insurance system to protect the health of uninsured Mexican migrants living and working in the USA, their families who remain in Mexico, and migrants who later return to Mexico [5,6]. China established social health insurance for rural-to-urban internal migrants, which helped reduce out-of-pocket payments (OPPs) for health services and removed the financial burden of inpatient services for health facilities [7].

Undocumented/illegal migrants often stay underground as they face many language, legal, cultural, and bureaucratic barriers when trying to access health care services. Addressing migrant health in any country is a complicated task as efforts always operate in a highly political sphere; these political influences ultimately shape the laws and regulations around the design of the health care system. Providing health care for undocumented/illegal migrants is therefore an even more complex and sensitive matter. Formally extending health care services to undocumented/illegal migrants could mean that the government agrees to bear the burden of health care costs, as well as accept the existence of migrants. It could also imply that the enforcement of national immigration laws is too weak to halt the influx of undocumented/illegal migrants. Nevertheless, many countries have developed either state-run or private initiatives and measures to create safeguards for such migrants and strategies vary by country. For example, in Philadelphia, a non-profit organization called “Puentes de Salud” provides health care and social services at low cost for illegal Latinos who are excluded from the Affordable Care Act (ACA) of the USA [8]. In certain EU countries, such as Germany and Italy, undocumented/illegal migrants are allowed to enjoy health care services with a varying range of benefit packages [9]. Some countries, such as France and Italy, allow certain vulnerable migrant groups of children and pregnant women to enjoy health benefits to the same extent as nationals, while some countries, like the UK and the Netherlands, do not permit this [9]. In Hong Kong, undocumented/illegal migrants are allowed to access health care services only in emergency situations [10].

In moving towards Universal Health Coverage (UHC), several countries in the Greater Maekong Subregion (GMS) have expanded health coverage to migrants, tackled barriers which restrict access to health services, and strengthened partnerships between key concerned stakeholders to improve migrant health along borders [11]. However, in most countries, policies to address the health needs of undocumented/illegal migrants have not been developed. Thailand is one of the GMS countries with distinct policies to protect the health of all migrants, including the introduction of health insurance policies since 1979 (First Immigration Act) and strengthening the provision of migrant-friendly services [12]. Thailand has been praised in the global health arena as one of very few countries in the world to have made concrete efforts to include undocumented/illegal migrants in national insurance coverage [13]. Policies for protecting migrant health (for both documented or undocumented migrants) in Thailand have, however, fluctuated over time under different government administrations. Currently, Thailand has two health insurance schemes under which migrants can access health services. (1) The Social Security Scheme (SSS), which is open for registered migrants typically employed in medium-size or large companies in the formal sector; and (2) the Compulsory Migrant Health Insurance (CMHI), managed by the Ministry of Public Health (MOPH), which targets other migrants, typically in the informal sector. Enrollment in either of the two schemes is required for migrants to obtain work permits and legal stay. However, migrant health insurance in Thailand has faced many problems. For example, the voluntary nature of migrant health insurance has been associated with adverse selection, with sick people tending to enroll more while the healthier migrants self-exclude. In addition, the operation of the CMHI under different government administrations (through different public hospitals) appears to be varied and does not sometimes adhere to the regulations; for example, although not common, it has been observed that unregistered migrants are sometimes allowed to buy the CMHI through some public hospitals. In sum, although Thailand has made progress to address migrant health over a long period of time, significant numbers of undocumented/illegal migrants have generally been unable to enroll in the Government health insurance scheme, resulting in smaller financial risk pooling [12,14].

### Background Information of the Migrant Fund

The Migrant Fund (M-Fund) is a voluntary, low-cost, non-profit health insurance scheme that has been designed to reach migrants uncovered by existing government insurance schemes in Thailand. The M-Fund is initiated and implemented by a private social enterprise, called ‘Dreamlopments’. Protecting undocumented/illegal migrants can be politically sensitive, yet some Thai government health authorities and local public hospitals have supported the implementation of the M-Fund to address existing challenges of providing healthcare for unregistered migrants in their local area. This was for two main reasons: (1) The project appeared to do no harm; and (2) while there was still no clear policy direction for this vulnerable group of people, it would be a good learning process for all involved parties. The project was launched in September 2017, in the northern region in Tak province in collaboration with Thai health facilities—namely Mae Sot, Mae Ramat, and Phop Phra hospitals—affiliated to the Ministry of Public Health (MOPH). The M-Fund also collaborates with local non-governmental organizations (NGOs) that provide health and other services for migrants along the Thai–Myanmar border, including Mae Tao Clinic (MTC), Shoklo Malaria Research Unit (SMRU), and Help without Frontiers (HWF). While MTC provides a vast range of health services, SMRU mostly focuses on providing care for pregnant women and migrants with tuberculosis or malaria. When migrant health problems are beyond their management capacity, these two clinics refer patients for more advanced care to Mae Sot, Mae Ramat, and Phop Phra hospitals. The majority of referred cases from the two clinics are pregnant women with complications and those requiring cesarean sections. The M-Fund has expanded its collaboration with HWF in order to protect the health of migrant children in a pilot migrant school in Tak by offering a medical policy for migrant children priced at 75 THB (US$2.37), with a monthly co-payment between parents (50 THB = US$1.58) and HWF (25 THB = US$0.79) for each child.

The M-Fund is financially supported by UNICEF, the Initiative 5%, the Global Fund, and the European Union. At the beginning, the project used the ‘M-Fund Policy 1.0’, in which design and pricing were informed by a thorough review of epidemiological information, a research survey among migrants in Tak, and support from an international micro-insurance expert [15]. The M-Fund 1.0 comprised three medical plans: **Plan A** covering any hospital admissions (except cosmetics, traditional medicines, and admissions due to chronic diseases); **Plan B** covering both hospital admissions and outpatient department consultations; and **Plan C** an option extension of Plan B covering chronic diseases. Entry exclusion existed for people already diagnosed with cancer, tuberculosis, HIV, congenital abnormalities, and permanent disability. An individual paid 60 THB/month (US$1.89) for Plan A, 100 THB/month (US$3.14) for Plan B, and 150 THB/month (US$4.71) for Plan C, with a coverage ceiling of 60,000 Bath/year (US$1885) for Plan A and 100,000 THB/year (US$3142) for Plan B and C. After the first eight months of operation, an evaluation was carried out by the same micro-insurance expert that conducted the baseline assessment. The evaluation revealed significant adverse selection of members with a high proportion of pregnant members (35.2% of total members) mainly enrolled from the SMRU clinics and people with chronic diseases (15.2%); this resulted in high healthcare cost claims for admissions. Based on these findings, the medical plans were revised in order to increase the risk pooling and reduce adverse selection.

**Figure 1:**
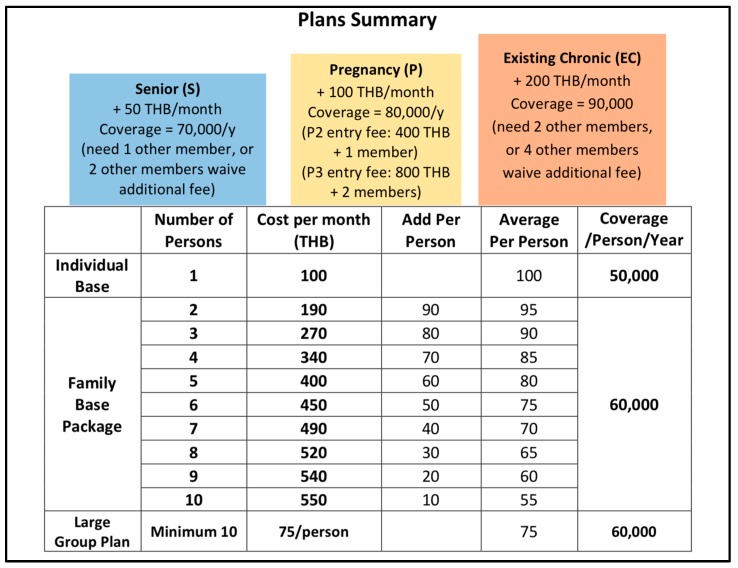
Summary of medical plans offered in the M-Fund Policy 2.0 (Source: M-Fund Policy 2.0, Dreamlopments).

The new medical model, ‘M-Fund Policy 2.0’, has been used since July 2018 and includes six plans or options: (1) An **individual base plan** is priced at 100 THB (US$3.14)/month and covers consultations and hospital admissions of an individual for up to 50,000 THB (US$1571)/year; (2) a **family base plan** involves an incremental 10 THB premium reduction for each additional family member and covers consultations and hospital admissions up to 60,000 THB (US$1885)/member/year; (3) a **large group plan** (when at least 10 people who are not from the same family enroll together) is priced at 75 THB/person/month and covers consultations and hospital admissions up to 60,000 THB (US$1885)/member/year; (4) a **chronic disease option** for those with chronic disease means people must enroll and pay an additional 200 THB (US$6.28)/month with extending coverage up to 90,000 THB (US$2828)/year, however, they are required to bring and maintain two additional members with them; (5) a **senior member option** for those aged 50 years and above, with an additional payment of 50 THB/month, provides coverage of up to 70,000 THB (US$2199)/year and requires an additional member; and (6) a **pregnancy option** for pregnant members costs an additional 100 THB (US$3.14)/month during pregnancy with coverage up to 80,000 THB (US$2513)/year. Additional conditions are that women enrolling during the second trimester of pregnancy (P2) must pay an entry fee of 400 THB (US$12.57) and bring an additional member, and women enrolling during the third trimester of pregnancy (P3) must add an entry fee of 800 THB (US$25.14) and bring two additional members. A summary of the medical plans, payments, and healthcare cost coverage in the ‘M-Fund Policy 2.0’ can be seen in Figure 1.

The cornerstone of the M-Fund operational approach is for community workers to provide information about the project to migrant communities and conduct community-based enrollment with the use of electronic tablets and a secure web application. Partner hospitals can view membership via a smart-phone scan of a Quick Response (QR) code (a matrix barcode that links to the M-Fund online system) provided to members, and the hospitals can directly bill the M-Fund for care provided via this QR code.

The M-Fund’s emphasis on providing health care to undocumented/illegal migrants presents a lesson worth learning for all countries seeking to achieve UHC. In the quest to achieve the Sustainable Development Goals and UHC and genuinely ‘leave nobody behind’ regardless of their citizenship and ethnic status, the M-Fund provides a strong example of the way forward.

Since the introduction of the M-Fund Policy 2.0, there has been real-time routine program indicator monitoring, but no systemic evaluation of program impact and its operational constraints. The objective of this study was to gather evidence and opinions from key stakeholders about the impact of the M-Fund on service users and healthcare providers, provide recommendations for areas which need improvement, and discuss the role of the M-Fund in assuring future health protection through informing the policy landscape and design of health insurance schemes for all migrants in Thailand.

## 2. Methods

### Data Collection

The study was conducted between December 2018 and March 2019, and employed a qualitative study design. Data were collected through three main techniques: (1) Document reviews relating to the M-Fund project in order to understand its background and context, such as the initial Feasibility Report, Health Micro-insurance for Migrants [16], evaluation report of the M-Fund Pilot Phase [15], M-Fund medical plans 1.0 and 2.0, and other documents in relation to micro-insurance and/or health insurance for migrants in Thailand and other countries; (2) in-depth individual and group interviews to obtain various perspectives on the project impact among three main groups, including: *(2.1) M-Fund partners:* International partners (Raks Thai foundation, WHO-Thailand); national partners (policy makers of the MOPH); and local networks (MTC, SMRU, and HWF) and services providers (Mae Sot, Mae Ramat, and Phop Phra hospitals) in Tak province; *(2.2) M-Fund clients*: Migrant patients and families, and *(2.3) M-Fund staff* in different positions and responsibilities; and (3) observations of activities involving the M-Fund and key partners to understand their relationships and practices.

The research team (NP, SJ, and PS) performed all the interviews and the International Health Policy Program provided internal training on how to perform the interviews and interact with the interviewees. The research team prepared and discussed semi-structured questions before going to conduct the fieldwork. The number of interviews was not limited initially, and the research team enrolled increasing numbers of participants when the fieldwork was ongoing until the interview data were saturated. Interviews with some M-Fund partners were conducted in Bangkok and in the MOPH, while the rest were carried out in Tak. The length of each interview lasted approximately 30 to 60 min. Interview information was recorded, transcribed, and triangulated with relevant documents. Inductive thematic analysis was performed through comprehensive considerations of important codes or phrases or condensed meaning units immerged during the interviews. The themes were constructed based on extensive discussions among the research team members during the fieldwork and directly after completion of the interviews. Limitations and challenges encountered by the M-Fund, and recommendations for addressing those challenges were investigated. The list of interviewees is shown in Table 1.

## 3. Results

Results are presented in two main sub-sections, firstly describing the situation of migrant health protection in Thailand and the characteristics of M-Fund members and secondly presenting the results from the thematic analysis.

### 3.1. General Situation Regarding Migrant Health Protection in the Fieldwork

#### 3.1.1. Overview of Health Insurance for Migrants in Thailand

In Thailand, there are three main health insurance schemes: (1) The Universal Coverage Scheme (UCS) for the general Thai population; (2) the Civil Servant Medical Benefit Scheme (CSMBS) for civil servants; and (3) the Social Security Scheme (SSS) for employees in the formal private sector [17]. All Thai nationals are insured for their health according to the Thai Constitution, while the situation of migrants’ health insurance has fluctuated over time depending on the politics in different time periods.

According to Suphanchaimat et al., migrant health policies in Thailand can be divided into four eras [14]. The first era (1900s to the 1990s) was the period when nationalism grew in response to the advent of colonialism in Southeast Asia. There was a special law (Por Wor 337) that repealed the Thai nationality of a person whose parents were non-Thai despite them being born within Thailand’s territory [18]. The second era began in early 1990s when increased industrialization aggravated a shortage of low-skilled labor, especially in difficult, dangerous, and dirty (3D) jobs. The government initiated amnesty policies to allow undocumented/illegal migrants to present to government authorities and obtain legal stay within a given period of time upon the issuance of a temporary work permit.

The third era (after 2004) started when undocumented/illegal migrants were required to participate in the ‘nationality verification’ (NV) process during windows of legal registration. During the NV process, migrants could acquire a temporary identity card from the Ministry of Interior (MOI) and a work permit from the Ministry of Labor (MOL) and were then granted a temporary passport as evidence of their legalized status. These registered migrants were, in principle, obliged to buy the yearly health insurance, ‘Health Insurance Card Scheme’ (HICS), of the MOPH. The card initially cost 1300 Baht (US$39) per year and increased to 2200 Baht (US$67) per year [19]. In 2013, the MOPH also initiated an insurance scheme for migrants’ dependent children under seven years old, charging 365 Baht (US$11) per year.

The fourth era (mid-2014 until present) was the period in which the government launched the ‘One Stop Service’ (OSS) to facilitate the registration of undocumented/illegal migrants. Migrants who failed to register with the OSS were subject to deportation. The price of a HICS card was reduced to 1600 Baht (US$48) per year for an adult, but the insured were required to pay 3200 Baht (US$96) for a two-year coverage [20]. In principle, all undocumented/illegal migrants need to be registered with the OSS, but some undocumented/illegal migrants still fail to register with the OSS, making them uninsured with the HICS at the same time. This phenomenon can be explained by reasons, such as: (1) Most undocumented/illegal migrants live in deprived conditions and are unable to afford the card price (this matter has become more complex in recent years as the MOPH increased the payment requirement to 3200 Baht (US$96) for a two-year coverage); and (2) as is the case with the general population, people do not want to be enrolled in health insurance when they are still healthy as they perceive that they may not have a chance to use it [21].

According to the recent Thailand migration report, there are a total of three million registered migrant workers through NV, MOU, and OSS from CLM countries and of these about two million workers are from Myanmar [22]. However, due to the limits of official data and the highly mobile characteristic of migrants, it is very difficult to provide an accurate estimated number of undocumented/illegal migrants (and their dependents) residing in the whole kingdom of Thailand.

#### 3.1.2. Migrant Behaviors along the Thai–Myanmar Border in Tak Province

**Figure 2:**
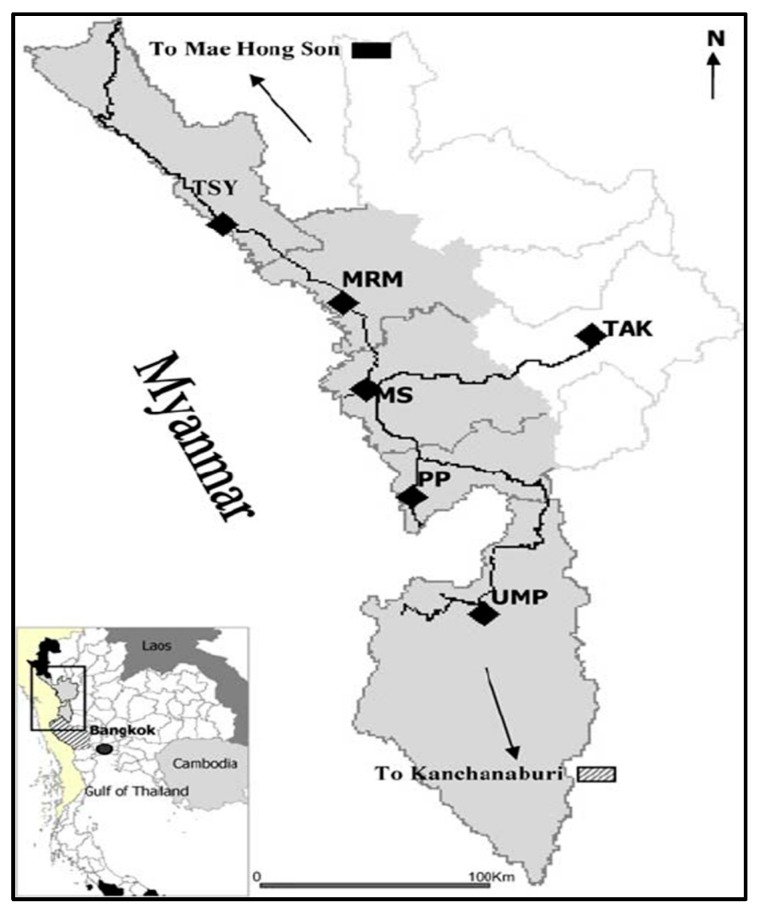
Map of the study area of the M-Fund project. Note: Thai districts along cross border in Tak include MRM = Mae Ramat, MS = Mae Sot, PP = Phop Phra, UMP = Umphang, TSY = Tha Song Yang. Source: https://www.researchgate.net/figure/Map-of-Tak-Province-The-study-area-is-shown-in-grey-DOI_fig1_7062209 (accessed 18 February 2019).

The M-Fund project has operated along the Thai–Myanmar border in Tak province, one of the most densely migrant-populated areas in Thailand. The map of the study area is shown in Figure 2.

The study area in Tak province comprises three districts, including Mae Sot, Mae Ramat, and Phop Phra. Mae Sot shares its border with Myawaddy township in Myanmar. The two areas, Mae Sot and Myawaddy, are separated from each other by the Moei River, but people from both countries can cross the Moei River easily. Many migrants cross the border from Myanmar to Thailand as seasonal workers or just to visit their relatives without any valid travel documents, including health insurance. 

Despite several strategies to address migrant health protection, gaps persist in systems and many migrants end up excluded from existing public health insurance schemes. This has a negative impact on the health of migrants but also has negative consequences for the financial status of public hospitals along the border. Many migrants, particularly undocumented/illegal migrants, visit hospitals only when they are severely ill and face costly treatment beyond their ability to pay. The unpaid debt is shouldered by the hospitals, leading to huge financial deficit.

#### 3.1.3. Characteristics of M-Fund Members

As of 19 March 2019, a total of 9131 people had enrolled as M-Fund members, including 2627 (28.8%) men and boys and 6504 (71.2%) women and girls. Among them, 3014 (33.01%) were inactive members who could not enjoy the benefits as they had stopped paying the premium and their membership had expired. The rest were active members able to enjoy the benefits and those who were waiting for the benefit activation (two weeks for new enrolments and three months for re-enrolments). The characteristics of the M-Fund members broken down by selected insurance packages are shown in Table 2.

Currently, enrolment into the M-Fund can be done at various sites, including in the community (such as at migrants’ houses and workplaces and an M-Fund drop-in center (DIC) in Mae Sot), MTC, Mae Sot hospital, and SMRU border migrant clinics. Most pregnant women register at the SMRU clinics, the M-Fund DIC, and MTC. The benefits for a pregnant member cover antenatal care (ANC) and delivery (either normal vaginal delivery or cesarean section, up to the coverage ceiling). Table 3 shows the number of pregnant women enrolled as M-Fund members at different trimesters of gestation, specifically at the SMRU clinics and in non-SMRU sites. Those enrolled through the SMRU outnumber those registering in the communities.

### 3.2. Results of Thematic Analysis

Two main themes emerged from the fieldwork and concern the positive value of the M-Fund project and the challenges it faces going forward. This analysis is based on perspectives of key stakeholders.

#### 3.2.1. Theme 1: The Positive Value of the M-Fund Project

**(1) Increased Access to Health Care Services for Undocumented/Illegal Migrants**

All interviewees agreed that the M-Fund project significantly helped provide health care for undocumented/illegal migrants. In Tak, migrant workers and their relatives are commonly ineligible to purchase the HICS as many are self-employed and cannot get work permits. With the M-Fund card, they can access health services at public hospitals along the border when needed.

“They (migrants) can receive health care treatments free of charge that even some Thai people may not be able to. It is super worth paying for.”—MF1

The advantages of the M-Fund card include: (1) The card can be sold to any migrant regardless of their registration status; (2) the medical plan is affordable and migrants can pay on a monthly basis (the HICS always requires a lump sum payment); and (3) all family members are eligible to purchase the card, independent of their work status.

**(2) Reduced Financial Burden for Healthcare Providers**

Hospitals participating in the project can reduce the unpaid debt they previously shouldered as a result of providing care for unregistered/uninsured migrants. One example from a hospital showed that, before joining the M-Fund, the hospital ran an annual deficit of about 60 million Baht (US$1.9 million). After the M-Fund came in, the deficit decreased to around 52 million Baht per year (US$1.6 million), an 8-million-Baht (US$250 thousand) improvement.

“We generally shoulder the social welfare cost (unpaid debt) by about 60 million Baht a year due to providing free services to (uninsured) migrants, but now it is reduced to around 52 million Baht a year, which might be because of the M-Fund.”—MP5

It is important to note that the cost reduction might not be solely due to the M-Fund. Other factors might have played a role, such as fewer visits/admissions of migrants, fewer complicated health problems among migrants, and fewer referred migrant patients. Moreover, one hospital director pointed out that the financial benefit from the M-Fund was not that significant, as previously, the hospital charged the costs of providing migrant health services to local NGOs. Then, later, the M-Fund covered these costs. Therefore, the NGOs benefitted most from the project, not the hospitals.

“Previously, we had NGOs responsible for paying the cost of health care services for some (uninsured) migrants. However, now the cost of those services has been paid by M-Fund, particularly for the referred cases. So, this doesn’t mean we get more money, it is just a change of who pays the money.”—MP6

**(3) Improved Referral Systems for Migrants**

Local NGO clinics cannot perform advanced medical care, such as cesarean sections or appendectomies, and mostly they refer severely ill patients to Mae Sot Hospital. The situation is more pronounced for pregnant women requiring caesarian sections. Interviewees in these clinics emphasized that due to the M-Fund, they can refer pregnant women to Mae Sot Hospital more easily and without serious concerns over the healthcare charges. The M-Fund deals directly with the hospital’s financial staff to handle the costs.

“We have to refer them (migrants) to the hospital because they have complicated health problems in about 5 percent of cases. So, for the 95 percent, we can manage by ourselves, but the rest 5 percent we need to refer. The cost for these 5 percent patients may be up to 4 million Baht per year…. …many of them are cesarean-section cases. The problem is, in Thai hospital, there’s always a charge. Very expensive. One normal cesarean-section causes us around 15,000 or 20,000 Baht.”—MP9

**(4) Protected Health for Migrant School Children**

A collaborative project between the M-Fund and a pilot migrant school in Tak was initiated in November 2018 and 58 school children enrolled in the scheme. Incidentally, a measles outbreak hit the school in December and two affected students who had enrolled in the M-Fund received free treatment in the partner hospital under the M-Fund coverage. School teachers and parents observed the benefits of being insured and this led to a second wave of registration with the M-Fund. As of March 2019, a total of 221 school children had enrolled in the M-Fund insurance scheme.

“There are always problems with school children. For an emergency case, we have to transfer them to Mae Ramat Hospital or Mae Sot Hospital, which is very costly. So I think this project is beneficial for children.… previously, whenever the children had accidents or health problems, the school had to take care of the cost, but having M-Fund helps us a lot.”—MP12

**(5) Increased Knowledge and Awareness about Migrant Health**

Significant numbers of migrants are not eligible to buy the HICS if they fail to register with the OSS. They are very mobile and live in deprived areas, which makes it difficult for them to access health services or pay the insurance premium. Furthermore, many of them do not understand what health insurance is and some are not willing to pay the premium because they are still healthy. The M-Fund has, to some extent, indirectly empowered and educated migrants about the concept of health insurance.

*“I would like to participate in the M-Fund project. Previously, I had a MOL card (as a worker) and so had health insurance too, but I had never been sick or visited hospital at all. But I would still like to have an M-Fund card, in case that I get sick or a health problem. If I have health problems, I would feel comfortable, but if I don’t have any health problems, it would mean that I make a merit in helping other people (those that get sick can use this pooled fund).”–MG6*

Table 4 summarizes perspectives among various stakeholders about the impact of the M-Fund. All key partners shared similar views that the project helped increase access to care for migrants. Policy makers and public hospital staff perceived that the project had helped reduce the hospitals’ financial burden. Local clinics were pleased that the M-Fund could partially support the healthcare cost of referred cases for advanced treatment at public hospitals. Migrant parents wished to have their children insured in order to receive proper healthcare services when needed. However, some migrants were still not aware of the concept of health insurance and why they need to pay an advance premium if they are healthy.

#### 3.2.2. Theme 2: Negative Aspects and Challenges for M-Fund

The M-fund clearly has several positive aspects, but it has also introduced complexity to the Thai health system. The current political direction intends to harmonize the three main health insurance schemes (CSMBS, SSS, and UCS). If the MOPH supported the advent of private micro-insurance, like the M-Fund, this would possibly contradict this overarching political direction. The quote below from one of the interviewees (MP2) underpinned this point.

“The issue is that the M-Fund somehow complicates the health care service systems with several cascades of insurance schemes and this could make the whole system fragmented”—MP2

The challenges that the M-Fund faces mainly relate to concerns about its sustainability, illustrated as follows.

▪ The M-Fund has not yet been able to recruit as many members as expected. In the feasibility study model, it was projected that in order to move towards financial autonomy, the M-Fund should have about 15,000 members by the end of year 1; 33,000 members by the end of year 2; and 50,000 members by the end of year 3 [16]. However, to date (as of 19 March 2019), more than a year after its start, the total number of members was just above half of the expected number for the first year, with a little over 9000 migrants enrolled.
▪ It is very difficult for the M-Fund to avoid the problem of adverse selection as long as purchasing the insurance card remains voluntary.
▪ The payment of 100 Baht/month (individual package) may be too cheap from the insurer’s perspective. The concept of group enrolment was introduced, such as with the ‘family package’, the ‘chronic disease option package’, and the ‘senior option package’, with some discounts on the premium. This aimed to increase the number of overall members and have a greater proportion of healthy members. Although this had a positive impact on enrollment levels, it led to another problem, as some individuals face difficulties in persuading other healthy people to enroll together as a group. The demand is also difficult for migrants who live alone or live remotely from the community.
▪ There is still a gap between the revenue gained from the premium collection and the healthcare reimbursements claimed by healthcare providers. Also, the M-Fund does not have any mechanisms to negotiate with the service providers to contain their treatment cost or to check if the treatment cost is clinically and financially appropriate.
▪ Financial support from donors is unpredictable. Although the project has received support through grants from UNICEF, the Global Fund through Raks Thai Foundation, the European Union, and the 5% Initiative, long-term donor support is not guaranteed in the event that the scheme does not become self-sustaining.

## 4. Discussion

Overall, the M-Fund project has been viewed as a helpful initiative to protect the health of migrants, who are poor and have precarious legal status. Based on key stakeholder perspectives, the M-Fund has contributed to improving migrants’ access to care and referrals to advanced care, including for children in migrant schools, raising awareness on migrant health protection, and reducing the financial burden for public hospitals. However, unsurprisingly, this initiative faces several challenges.

To be sustainable in the long term, the most important task for the M-Fund is to increase community engagement to encourage better risk-sharing or increase the pooled fund. The M-Fund’s characteristics are relatively close to those of other CBHIs. They typically operate on a voluntary basis and are managed by community-based organizations, other than government or a private for-profit company, and provide risk-pooling to cover the costs (or some parts) of health care services [2]. The voluntary membership aspect makes the CBHIs prone to adverse selection, with an imbalanced proportion of sick members and healthy members, leading to high health care costs beyond the revenues generated from the collected premiums [23]. Thus, the M-Fund needs to ensure sufficient risk pooling which allows for the transferring of funds from low-risk to high-risk members and from wealthier to poorer members. The pooled fund collected should provide adequate financial protection of those households who need it most, which can be achieved by using various strategies.

Firstly, increasing knowledge and awareness of health insurance and the idea of risk- and responsibility-sharing among migrants is essential. A systematic review of CBHIs in Southeast Asia suggested that involving the community was an important step to improve the management of such schemes [3]. Promoting health insurance as a welfare scheme to communities as a whole, with the support of migrant peers, community leaders, and religious leaders, might be a good strategy for the M-Fund in order to encourage migrants to enroll in the scheme before they get sick. Findings also showed that although the undocumented/illegal migrant status per se might prevent people from enrolling on the scheme, this was not the case for the M-Fund and such a notion did not emerge during the study period. This may be because the M-Fund is implemented by an independent non-profit social enterprise, where the community workers who provide information to migrant communities and conduct enrollment are trusted Thai-Burmese peers who are themselves living in migrant communities. In addition, some M-Fund members enrolled when they needed treatment for certain health conditions and therefore their greatest concern is to receive health care rather than worry about being arrested by the police. Furthermore, the Myanmar people in the study had relatively similar ethnic backgrounds, had a good sense of community, and were happy to help their migrant peers benefit from the scheme (see results section, theme 1: No. 5).

Secondly, extending the project to other geographical areas can help improve risk pooling as the volume of enrollees increases. Based on interviews with the M-Fund management team, work is underway to extend the program in Myawaddy township in Myanmar, as well as other border areas of Thailand, such as in Sakaeo province along the Thailand–Cambodia border in the east. Regardless of where the project is expanded, a few important issues need to be considered. The M-Fund should identify appropriate healthcare providers to suit migrants’ health-seeking behaviors in any given area. Generally, migrants are willing to visit local clinics rather than well-established health facilities, like public hospitals. Therefore, it is necessary to identify potential facilities where most migrant patients tend to visit. A feasibility study should be conducted to understand the specific context of the new area before its expansion. Novel strategies and innovations to effectively trace migrants’ mobility should be further explored. As most migrants are highly mobile, mobile and Global Positioning System (GPS; an interconnected system of satellites and receivers that allows for the precise pinpointing of locations anywhere on or directly above the earth) technologies may be useful, not only for collecting premiums but also for disease protection and health promotion among migrants. In addition, collaborating closely with community leaders to provide more proactive education about the concept of insurance is required. This will help change the mindset of community residents that being insured means not only individual health protection, but protection for communities as a whole, which will in turn help mitigate the problem of adverse selection.

Thirdly, introducing additional benefits may help incentivize members to maintain their membership and increase risk pooling. For example, the M-Fund may grant rewards or discounts to long-term members who have never made any healthcare claims. The M-Fund is now planning to introduce ‘M coins’ to be gained from monthly premium contributions and deducted from the use of health services, to incentivize healthy members to stay in the program and not overuse services.

All recommendations above are important elements for the stability or sustainability of the M-Fund per se. However, it is also important for the M-Fund to maintain awareness of and be ready to adapt itself to the dynamics of migrant policies in Thailand. Many public health authorities have shown ‘written’ commitment to protect everybody’s health in Thailand. The National Health Security Office (NHSO), the main governing body of the UCS, clearly stipulates in its vision that ‘Everyone on the Thai soil is protected by Universal Health Coverage with confidence’ [24]. The MOPH endorsed a ‘Master Plan for Solving and Developing Public Health at Borders (2012–2016)’, indicating it is committed to protecting the health and quality of life for all people in Thailand regardless of their citizenship or immigration status [25]. In consideration of these commitments, the M-Fund could be supported as an insurance alternative to cover uninsured migrants. However, it is not yet fully clear if the MOPH views the M-Fund as an ally in its mission to cover the health of all migrants, or a competitor that contradicts or confuses policy orientations. Furthermore, support of the M-Fund from the MOPH could be further interpreted as accepting the presence of undocumented/illegal migrants, contradicting the supreme political direction. The M-Fund should continue to engage with health and government authorities, to increase recognition of its potential value, not only as an existing safety net for migrants who remain uninsured but also through the lessons it can offer to inform future design of government insurance schemes for migrants. Direct outreach to migrant communities, aligning premium collection with revenue streams of migrants and using digital systems and portability of coverage, appear to be particularly suited for this vulnerable and mobile population.

This evaluation has some limitations. First, the information shown in this study is mainly based on participants’ perspectives, which might be prone to bias under investigation. However, as this study employed a qualitative approach, all information derived from each participant was triangulated with interview findings from other informants, a document review, and researchers’ observations. Second, the study period lasted around two weeks, which is quite a short and limited time-span to be able to fully identify the dynamics of the migrant situation in Thailand or in Tak specifically. However, we believe that the information shown here is rich enough to provide meaningful information to all relevant stakeholders, including the M-Fund. Third, information about CBHI schemes, particularly those targeting undocumented/illegal migrants in particular, is very limited. This makes it quite difficult to provide more intensive discussion on the interaction between CBHI and undocumented/illegal migrants. This issue points to a critical gap in knowledge in the migrant health field and warrants much further research.

## 5. Conclusions

Despite covering a small number of migrants in Thailand, the M-Fund has demonstrated several positive values, including improving access to health care for previously uninsured migrants, raising awareness on migrant health protection, and reducing the financial burden for public hospitals in the project area. To make it sustainable and more effective, the M-Fund may have to make a greater effort to increase the size of risk sharing and reduce adverse selection. It also needs to reduce its operational costs, explore novel technologies in collecting premiums, and work even more closely with the community to help migrants understand the benefits of being insured when they are healthy. Expanding the project to other areas is worth pursuing but depends upon strong planning to address any future challenges. For example, the specific contexts of different borders (such as differences in migrants’ health-seeking behaviors and their level of knowledge and awareness of health insurance) and dynamic changes of the political atmosphere need to be taken into consideration. While there is no clear policy direction on health protection for undocumented/illegal migrants, the M-Fund appears to be a good safety-net initiative for those who are left behind. Continued engagement with policy makers is important to gain further recognition and support or to inform the future design of stronger government schemes for this population.

## Figures and Tables

**Table 1:** List of interviewees.

Code	Date	Role	Involvement with M-Fund
**Partners**			
MP1	28 January 2019	Policy maker, MOPH	Support collaboration with local MOPH
MP2	28 January 2019	Policy maker, MOPH	Provide advice during the design
MP3	25 January 2019	International development partner	Provide some financial support
MP4	31 January 2019	International development partner	Provide advice during the formulation
MP5	4 February 2019	Hospital director A	Provide services for M-Fund users
MP6	6 February 2019	Hospital director B	Provide services for M-Fund users
MP7	7 February 2019	Hospital director C	Provide services for M-Fund users
MP8	5 February 2019	Local partner	Financial support for the school project
MP9	5 February 2019	Local partner	Provide services for M-Fund users
MP10	5 February 2019	Local partner	Provide services for M-Fund users
MP11	6 February 2019	Local partner	Provide services for M-Fund users
MP12	6 February 2019	Local partner	Collaborate with the school project
**Clients**			
MG1	5 February 2019	Inpatient at Hospital A	M-Fund beneficiary admitted at a public hospital
MG2	5 February 2019	Inpatient at Hospital A	M-Fund beneficiary admitted at a public hospital
MG3	5 February 2019	Migrant family and friends *	M-Fund beneficiaries in local community
MG4	6 February 2019	Migrant family and friends *	M-Fund beneficiaries in local community
MG5	6 February 2019	Migrant family and friends *	M-Fund beneficiaries in local community
MG6	7 February 2019	Migrant family	M-Fund beneficiaries in local community
**M-Fund officers**			
MF1	5 February 2019	M-Fund community worker	Work as a Community Worker at project information and registration counter in a public hospital
MF2	5 February 2019	M-Fund community volunteer	Work as a Volunteer in local community
MF3	5 February 2019	M-Fund staff at the office	Work as a Project Manager
MF4	5 February 2019	M-Fund community workers (3) *	Work as Community Workers at drop-in center in local community
MF5	6 February 2019	M-Fund community workers (2) *	Work as Community Workers in local community
MF6	8 February 2019	M-Fund staff at the office	Work as a Medical Officer
MF7	8 February 2019	M-Fund staff at the office	Work as a Director

Note: * Group interview.

**Table 2:** Characteristics of M-Fund members by types of selected plan options.

Status	Chronic	Senior	School	Others	Total
Active	1608 (69.9%)	1171 (81.7%)	208 (94.1%)	3130 (60.5%)	6117 (66.99%)
Inactive	693 (30.1%)	263 (18.3%)	13 (5.9%)	2045 (39.5%)	3014 (33.01%)
Total	2301 (100%)	1434 (100%)	221 (100%)	5175 (100%)	9131 (100%)

Note: (1) M-Fund members as of 19 March 2019, (source: M-Fund). (2) Chronic = members who enrolled with the chronic disease option, Senior = members who were 50 years and above and were under the senior option, School = children enrolled under the school plan, Others = other members (including pregnant women) who did not fall into the first three categories.

**Table 3:** Pregnant members enrolled in M-Fund project.

Status	1st Trimester		2nd Trimester		3rd Trimester		Total	
	Non-SMRU	SMRU	Non-SMRU	SMRU	Non-SMRU	SMRU	Non-SMRU	SMRU
Active	172 (93.5%)	293 (85.9%)	187 (85.0%)	265 (82.0%)	47 (79.7%)	108 (65.5%)	406 (87.7%)	666 (80.3%)
Inactive	12 (6.5%)	48 (14.1%)	33 (15.0%)	58 (18.0%)	12 (20.3%)	57 (34.5%)	57 (12.3%)	163 (19.7%)
**Total**	184 (100%)	341 (100%)	220 (100%)	323 (100%)	59 (100%)	165 (100)	463 (100%)	829 (100%)

Note: (1) M-Fund pregnant members registered from 1 July 2018 to 16 March 2019 (source: M-Fund). (2) SMRU = Shoklo Malaria Research Unit. (3) Enrolment in communities and other locations were combined as non-SMRU.

**Table 4:** Perspectives of stakeholders on degrees of positive impact of the M-Fund.

Key Partners	Increased Access to Care for Migrants	Reduced Financial Burden for Health Providers	Improved Referral Systems for Migrants	Protected Health for School Children	Increased Knowledge and Awareness of Migrant Health
MOPH	+++	+++	++	n/a	++
Public hospitals	+++	+++	++	n/a	++
SMRU	+++	++	+++	n/a	++
MTC	+++	++	+++	n/a	++
HWF	+++	++	+++	+++	++
Migrant patients	+++	n/a	n/a	++	+
Migrant families	+++	n/a	n/a	++	+

Note: +++ = Very positive, ++ = Positive, + Somewhat positive, n/a = Not applicable.
